# Trends of obesity and overweight among children and adolescents in China

**DOI:** 10.1007/s12519-023-00709-7

**Published:** 2023-03-15

**Authors:** Ye Hong, Rahim Ullah, Jian-Bing Wang, Jun-Fen Fu

**Affiliations:** 1 Department of Endocrinology, Children’s Hospital, Zhejiang University School of Medicine, National Clinical Research Center for Child Health, National Children’s Regional Medical Center, Hangzhou, 310052 China; 2 Zhejiang University School of Medicine, Hangzhou, 310052 China

**Keywords:** Adolescents, Children, Obesity, Overweight, Policy

## Abstract

**Background:**

Recent decades have shown a rapid increase in the prevalence of overweight and obesity among Chinese children based on several national surveys. Restrictions due to the coronavirus disease 2019 outbreak have worsened its epidemiology. This review updates the trends in the prevalence of overweight and obesity among Chinese children and adolescents and analyzes the underlying reasons to provide evidence for better policy making.

**Methods:**

Studies published in English and Chinese were retrieved from PubMed, Google Scholar, China National Knowledge Infrastructure and Wanfang.

**Results:**

The prevalence of overweight and obesity has been increasing for decades and varies with age, sex and geography but is more pronounced in primary school students. The increase in obesity in boys appeared to be slower, whereas that in girls showed a declining trend. The northern areas of China have persistently maintained the highest levels of obesity with a stable trend in recent years. Meanwhile, the prevalence in eastern regions has dramatically increased. Notably, the overall prevalence of obesity in children has shown a stabilizing trend in recent years. However, the occurrence of obesity-related metabolic diseases increased. The effect of migrants floating into east-coast cities should not be neglected.

**Conclusions:**

The high prevalence of overweight and obesity among Chinese children and adolescents persists but with varying patterns. Obesity-related metabolic diseases occur more frequently despite a stable trend of obesity. Multiple factors are responsible for the changing prevalence. Thus, comprehensive and flexible policies are needed to effectively manage and prevent the burden of obesity and its related complications.

## Introduction

In recent years, China has experienced rapid economic growth and urbanization, which is associated with an increase in obesity and a decrease in stunting and thinness among Chinese children [1–4]. There has been a general increasing trend of obesity in Chinese children and adolescents. In particular, the prevalence of obesity in children and adolescents aged 7–18 years was 0.1% in 1985 and increased to 7.3% in 2014 according to the Working Group on Obesity in China for diagnosing obesity [5] and 6.4% according to World Health Organization standards [3]. Consequently, obesity has raised concern owing to its threat to children’s health from other associated diseases, such as diabetes, cardiovascular diseases, and non-alcoholic fatty liver diseases (NAFLD) [6–9]. Additionally, obesity is an impactful factor of precocious puberty among children and adolescents [10]. As obese children are more likely to maintain obese phenotypes in later life [11], timely monitoring of the status of obesity in children and adolescents is crucial.

Several national surveys have been conducted to achieve dynamic surveillance of children’s obesity and overweight (Table 1). The Chinese National Survey on Students’ Constitution and Health (CNSSCH) was composed of seven cross-sectional surveys and conducted between 1985 and 2014. The China National Nutrition Survey (CNNS) was composed of five cross-sectional surveys and conducted between 1959 and 2012. The children and adolescents included in the CNSSCH and CNNS were from all subnational provinces, autonomous regions, and municipalities in mainland China. Meanwhile, the China Health and Nutrition Survey (CHNS) was composed of ten cross-sectional surveys and conducted between 1989 and 2015. This survey selected 15 regions in Chinese mainland with some regions included in later periods. All three national surveys utilized a multistage stratified clustering sampling method for participant recruitment. Despite the addition of the first cycle (2015–2019) of the China Chronic Disease and Nutrition Surveillance survey, which combined the CNNS and China Chronic Disease and Risk Factor Surveillance (CCDRFS), data on recent health surveillance among children and adolescents remain limited.

**Table 1:** Summary of national studies on obesity and overweight in Chinese children and adolescents

Author, year	Sample size	Time period	Age (y)	Criteria
Prevalence and risk factors for obesity and diabetes in youth study				
Zhang et al., 2021 [12]	201,098	2017–2019	3–18	(BMI_24_, BMI_28_) [28]
Yuan et al., 2021 [13]	14,597/14,597	2009/2019	6–15	BMI (WHO)
Chinese national survey on students’ constitution and health				
Ji et al., 2009 [14]	409,946/145,442/204,966/209,454/226,602	1985/1991/1995/2000/2005	7–18	BMI (WGOC)
Ma et al., 2012 [15]	204,977/216,786/234,421/215,319	1985/1995/2000/2005/2010	7–18	BMI (WGOC)
Song et al., 2013 [16]	68,420/70,426/39,204/38,940/47,043/35,886	1985/1991/1995/2000/2005/2010	7–18	BMI (WGOC)
Song et al., 2016 [17]	Total 1,280,239	1985/1995/2000/2005/2010	7–18	BMI (WHO, IOTF)
Wang et al., 2017 [5]	409,946/204,977/216,786/234,421/215,319/214,354	1985/1995/2000/2005/2010/2014	7–18	BMI (WGOC)
Jia et al., 2019 [18]	No direct report	1985/1995/2000/2005	7–18	BMI (WGOC)
Dong et al., 2019 [3]	409,836/204,763/216,073/234,289/215,223/214,301	1985/1995/2000/2005/2010/2014	7–18	BMI (WHO)
Dong et al., 2019 [4]	204,932/209,167/225,213/208,136/207,154	1995/2000/2005/2010/2014	7–18	BMI (WHO)
Song et al., 2019 [2]	409,836/204,754/214,281/232,008/215,184/213,890	1985/1995/2000/2005/2010/2014	7–18	BMI (WHO)
Dong et al., 2019 [19]	107,741/107,473	2010/2014	7–12	BMI (WGOC)
China health and nutrition survey				
Zhang et al., 2010 [20]	1977/2048/2350/1872/1275	1991/1993/1997/2000/2004	6–18	BMI (IOTF)
Cui et al., 2010 [21]	2581//2392/2389/2290/1463/1174	1991/1993/1997/2000/2004/2006	7–17	BMI (WGOC, IOTF)
Zhang et al., 2018 [22]	1458/1084	2011/2015	7–18	BMI (WGOC, WHO, IOTF)
Ma et al., 2021 [23]	11,985	1993–2015	6–17	WC & WHtR
China national nutrition survey/China national nutrition and health survey				
Ma et al., 2005 [24]	78,704/209,849	1992/2002	0–6 7–17	BMI (WHO) BMI (WGOC)
Wu et al., 2006 [25]	209,849	2002	0–6 7–17	BMI (WHO) BMI (WGOC)
Li et al., 2008 [26]	10,127/15,501/44,880	1982/1992/2002	7–17	BMI (IOTF)
Yu et al., 2018 [27]	32,862	2010–2013	0–5	BMI (WHO)

*WHO* World Health Organization, *WGOC* Working Group on Obesity in China, *IOTF* International Obesity Task Force, *WC* waist circumference, *WHtR* waist-to-height ratio, *BMI* body mass index

The coronavirus disease 2019 (COVID-19) outbreak has had a considerable impact on obesity [29]. An increased prevalence of obesity among Chinese youth during the COVID-19 pandemic has been reported [30–34]. In this review, we comprehensively summarize the national surveys and latest studies on the prevalence of overweight and obesity among Chinese children and adolescents and update the pattern in relation to age, sex and geography in recent years. After discussing the possible reasons for the changes, we outline the measures taken by the Chinese government and institutions for better management of obesity and overweight in the young generation. Finally, we appeal for a cooperative network among governments, families, schools, and health institutions.

## Methods

Using various combinations of the following search terms: “prevalence”, “obesity”, “overweight”, “children”, “adolescents”, “national surveys”, “risk factors”, “complications”, “policy”, “Chinese”, and “China”, we searched PubMed, Google Scholar, China National Knowledge Infrastructure and Wanfang for original articles and reviews published in English and Chinese between January 2001 and December 2022. We selected national survey-related literatures by reviewing their title, abstract and full text. Subsequently, the reference lists of the selected papers were reviewed. We excluded studies on single regions. Additionally, we used the search engine Baidu and the official websites of governments for related policies and principles.

## Results

### Trends of obesity and overweight in the past decades in China

Several national survey-based studies have consistently reported the increasing trend of obesity and overweight among Chinese children and adolescents over the last three decades. According to CNSSCH data, the mean body mass index (BMI) of children and adolescents aged 7–18 years increased from 17.0 kg/m^2^ in 1985 to 17.5 kg/m^2^ in 1995, 18.2 kg/m^2^ in 2005, and 19.0 kg/m^2^ in 2014 [3]. The prevalence of overweight and obesity was 1.1% and 0.1% in 1985 and 12.1% and 7.3% in 2014, respectively [3, 5], consistent with the reports from the CNSSCH in different time periods [15, 17]. The CNNS and CHNS also revealed similar trends of increased BMI, overweight, and obesity among children and adolescents over the past decades [20, 21, 23, 24, 26]. Studies have been conducted to update these statistics in younger generations for better prevention and management (Table 1).

### Changes by age

For children aged < 6 years, the prevalence of overweight and obesity increased from 2.3% and 1.6% in 1992 to 3.4% and 2.0% in 2002 and 8.4% and 3.1% in 2010–2012, respectively [24, 27]. However, in the China Chronic Disease and Nutrition Surveillance survey (2015–2019), which combined the CNNS and CCDRFS, the prevalence of overweight and obesity in this age group was 6.8% and 3.6%, respectively [35], showing a decreased trend for the prevalence of overweight in comparison with that in 2012. Separately, Gao et al. examined national surveys and found a similar decrease in the prevalence of both overweight and obesity in preschool children [36].

The prevalence of overweight and obesity in 6–18-year-old participants increased as reported by national surveys [3, 5, 15, 20]. In one CHNS study, the prevalence of overweight increased from 6.5% to 16.1% in children aged 6–11 years and from 3.3% to 6.2% in children aged 12–18 years from 1991 to 2004 [20]. Based on CHSSCH surveys, the prevalence of overweight and obesity in 2010 was 17.14% in primary school students aged 7–12 years, followed by 13.11% in junior school students aged 13–15 years and 10.88% in high school students aged 16–18 years [15]. The values for these three populations increased to 22.5%, 17.3%, and 15.4% in 2014 [5]. However, a later CHNS-based study found that the prevalence of overweight and obesity was stable in children aged 7–11 and 12–18 years from 2011 to 2015 [22]. The participants in this study were from 12 provinces, with 1458 individuals included in 2011 and 1084 in 2015, which is considerably lower than that in CHSSCH-based studies during the same study period (Table 1). Additionally, the geographical distribution of the participants is not comparable; 997 children were from the southern area, which is twice as many as those from the northern areas (*n* = 461). Consequently, this may induce bias during analysis. Another study reported a stable and slight downward trend of overweight and obesity among children aged 3–19 years [37]. Since over half of the children in this study were aged 3–6 years, it is difficult to reflect the real trend at all ages.

The recent Prevalence and Risk Factors for Obesity and Diabetes in Youth (PRODY) study, which included over 200,000 participants in 2017–2019, reported the highest prevalence of obesity in children aged 8–13 years [12]. Furthermore, this study compared data from two national multicenter surveys conducted from July 2009 to July 2010 and July 2017 to July 2019 [13]. Overall, 14,597 pairs of children and adolescents aged 6–15 years were recruited from four provinces or cities representing three main regions in northern, southern, and eastern China. Interestingly, the PRODY study found a continuously increasing trend of overweight among boys aged 6–14 years, with a pronounced increase at the age of 8–11 years. Among girls aged 6–10 years, there was no significant change in the prevalence of overweight or obesity, and their BMI standard deviation score (SDS) decreased from 2009 to 2019. The BMI SDS and the prevalence of overweight and obesity increased slightly but not significantly for girls aged 11–14 years [13]. Collectively, the increase in the prevalence of overweight and obesity for primary school students (around 7–12 years old) merits sufficient consideration, although a stabilized trend has emerged among Chinese youth.

### Changes by sex

The prevalence of overweight and obesity is higher in boys than in girls [5, 15, 38]. National surveys confirmed these trends in different time periods, with great disparities between boys and girls, irrespective of age, location, and economic status [5, 14, 16, 18, 39]. However, the PRODY study found a higher obesity prevalence in boys than in girls only in eastern and northern China in 2017–2019 [12]. By comparing data from 2009 to 2019, the PRODY study reported an increase in the prevalence of overweight and obesity in boys by 2.5% and 1.8%, respectively, with lower average annual increases in comparison with 2010–2014 [2, 5]. Obesity prevalence in girls decreased by 0.9%, whereas overweight prevalence increased by 1.5% in the same decade, with a lower annual increase compared with 2010–2014 [2]. Furthermore, the overall rate of overweight and obesity in girls was not affected [13]. A meta-analysis also reported a declining trend of overweight in young people [40]. The rising trends of overweight in boys and girls observed since 1991 peaked in 2006–2010 (prevalence of 16.0% and 10.3% for boys and girls, respectively), and both sexes showed declining trends in overweight (14.4% in boys and 9.1% in girls) from 2011 to 2015 [40]. Therefore, the prevalence of overweight and obesity in boys slows down and that in girls appears to be stabilized.

### Changes by region (North, South, West, and East China)

There are prominent geographical disparities. According to the CNSSCH data, the northern parts of China, including Beijing, Shandong, and Tianjin, had the highest prevalence of overweight and obesity in 1985. Conversely, Guangxi and Guangdong, in the west and south, respectively, showed the lowest prevalence. The northern provinces and cities continued to have the highest prevalence in 2005 [18]. In a more recent CNSSCH study (2010–2014) and the PRODY study (2017–2019), northern China maintained a higher prevalence of overweight and obesity [12, 19]. The PRODY study revealed an increase in overweight but no significant increase in obesity across all investigated regions from 2009 to 2019 [13]. However, each area had its own pattern, as shown in Table 2. The prevalence of obesity in the northern area (Beijing and Tianjin) showed a declining trend, whereas that of overweight continued to rise. Notably, the prevalence of both overweight and obesity in the eastern area dramatically increased during that decade. A decreasing trend in overweight and a consistent trend in obesity were observed in Guangxi. Therefore, the northern area, with the largest prevalence, warrants more attention for better control and prevention of overweight and obesity, whereas the eastern areas, with its rapid increases in both overweight and obesity, should take appropriate actions to slow down or stop further progression.

**Table 2:** Changes in the BMI, overweight, and obesity in Chinese children from 2009 to 2019 [13]

Areas	Total	North (Beijing & Tianjin)	East (Zhejiang)	South (Guangxi)
BMI SDS	No changes (*P* = 0.06)	No changes (*P* > 0.05)	Increase (*P* < 0.01)	Decrease (*P* < 0.01)
Overweight	Increase 2.0% (*P* < 0.01)	Increase 2.3% (*P* < 0.01)	Increase 3.8% (*P* < 0.01)	Decrease 1.9% (*P* < 0.05)
Obesity	No changes (*P* = 0.16)	No changes (*P* > 0.05)	Increase 3.1% (*P* < 0.01)	No changes (*P* = 0.10)

Data were authorized by Jin-Ling Wang et al. *BMI SDS* body mass index standard deviation score

### Comparison of urban and rural areas

Based on national surveys in China, the prevalence of overweight and obesity among young individuals is higher in urban than in rural areas [15, 19, 40]. According to CNNS, the prevalence of overweight and obesity in children and adolescents aged 6–17 years was 13.2% and 8.9% in large cities; 10.6% and 7.6% in medium-sized and small cities; 8.9% and 5.6% in common villages; and 7.5% and 4.3% in poor villages, respectively [38]. Both urban and rural areas showed an upward trend. The prevalence of obesity among Chinese urban children increased from 0.2% in 1985 to 8.1% in 2010 [16]. Compared with data in 2002, young individuals in cities showed a higher prevalence of overweight and obesity in 2012. However, the increase in rural areas was twice that in cities [41]. In particular, the average annual increase in overweight and obesity among rural children exceeded that among their urban peers in 2005–2010 [5]. Data from the CNSSCH also showed a decreasing disparity in the prevalence of overweight and obesity between urban and rural areas from 2010 to 2014, surpassing the overweight and obesity prevalence among rural children in several eastern areas in 2014 [19]. Consistently, a worse situation in preschool children from rural areas was projected [36]. In contrast, Guo et al.’s meta-analysis reported a decreased prevalence of overweight and obesity in both urban and rural areas after 2010 [40]. Due to fewer studies included in Guo et al.’s calculation for urban and rural areas in this period, the decreasing trends need to be consolidated in future studies.

### Obesity-related metabolic disorders among Chinese children and adolescents

Obesity is highly associated with multiple diseases, including NAFLD, metabolic syndrome and precocious puberty [9]. Although the prevalence of obesity and overweight showed a slowing or a stabilizing trend in children, the prevalence of complications of obesity continues to increase, jeopardizing the health of children and adolescents.

### Obesity and nonalcoholic fatty liver disease

NAFLD is a chronic liver disease characterized by excess fat deposition in the liver without other etiologies. Obesity is the largest risk factor for NAFLD, and the prevalence of NAFLD is rising along with the increase in childhood obesity [42]. The prevalence of NAFLD in American adolescents doubled in 2007–2010 compared with that in 1988–1994, from 3.9% to 10.7%. Meanwhile, the proportion of NAFLD in obese participants is higher for both sexes [43]. Similarly, the prevalence of NAFLD in Asian children increased from 4.42% before 2010 to 7.10% after 2010 [6]. Specifically, one meta-analysis indicated that the prevalence of NAFLD in Chinese children in 2000–2010 was 4.0% and increased to 7.7% in 2011–2021 [44]. Data from Hangzhou, Zhejiang Province, demonstrated that the NAFLD prevalence was as high as 57.6% in enrolled obese children, with no difference between 2008–2012 and 2013–2017 [45]. Nonalcoholic steatohepatitis (NASH) is an advanced stage of NAFLD, with apparent infiltration of immune cells and abnormal alanine aminotransferase and/or aspartate aminotransferase levels, resulting from damaged hepatocytes and subsequent abnormal liver function. Surprisingly, among children and adolescents with obesity, those who have abnormal liver function account for 60.4%, second only to acanthosis nigricans (AN) at 69.3%. Furthermore, the proportion of abnormal liver function significantly increased, from 68% in 2008–2012 to 78.6% in 2013–2017 in obese boys aged > 10 years [45]. Although not all patients who have abnormal liver function can be diagnosed with NASH, the authors emphasized the potentially damaged liver function resulting from excess fat accumulation in the liver in obese individuals. Although the increase in NAFLD in children is not fully dependent on the obesity rate, as reported by some studies [46, 47], it is critical to realize that obesity has a profound contribution to the NAFLD disease burden, and therefore, action is needed to strengthen diagnosis and intervention in clinical practice.

### Obesity and metabolic syndrome

The development of obesity is accompanied by other disorders in children and adolescents, including hypertension, dyslipidemia, and abnormal glycemia. To better recognize and evaluate the outcomes of the cluster of these disorders in youth, the International Diabetes Federation (IDF) proposed the definition of metabolic syndrome in children and adolescents in 2007 [48]. The Chinese Pediatrics Society published the definition of metabolic syndrome in 2012, which was fundamental for its prevention and control [49]. In a national study of 15,045 children from seven Chinese provinces, the prevalence of metabolic syndrome calculated using the IDF criteria was 2.3% [50]. The prevalence was up to 40.1% for obese children aged > 10 years [45].

Obesity due to overfeeding impedes glucose clearance by disrupting insulin signaling in the main metabolic organs, including the muscle and liver, which results in hyperglycemia [51]. Based on the criteria for diagnosing abnormal glycemia in Chinese children and adolescents [49], Wang et al. reported a remarkable increase in the incidence of abnormal glycemia from 26.5% in 2008–2012 to 39.1% in 2013–2017 in young obese boys and from 29.1% to 37.8% in older obese boys [45]. Similar to abnormal glycemia, hypertension is considered a symptom of metabolic syndrome [49]. Children with obesity are more likely to have elevated blood pressure [52–56]. Surprisingly, evidence showed that the incidence of hypertension in obese girls nearly doubled from 20% in 2008–2012 to 40% in 2013–2017 regardless of age [45]. The increase in hypertension in obese boys was also remarkable. This brings great challenges to dealing with hypertension and its related cardiovascular diseases, as obesity in children increases the risk of coronary heart disease in adulthood [57, 58].

AN is a visible abnormal skin manifestation on the neck or back, with high levels of pathological cell proliferation [59]. It is highly associated with insulin resistance, which usually causes hyperinsulinemia and abnormal glycemia, as well as hyperlipidemia and high blood pressure [60–62]. The AN prevalence in Chinese obese children is 69.3%, with a higher value in boys (71.9%) than in girls (64%) [45], which is similar to that in Iranian obese children (67.6%) [63]. Unlike the metabolic disorders mentioned above, dyslipidemia showed a distinct trend. A significant decrease in the incidence of dyslipidemia was reported after 2013 in boys with obesity [45], and a decreasing trend was also observed in all recruited girls, although it was not statistically significant.

### Obesity and precocious puberty

Precocious puberty, featured by early onset (before 8 years in girls and 9 years in boys) of secondary sexual characteristics [64], emerges as an obesity-related metabolic disease. Its prevalence in China has increased, as reported by multicenter studies [65, 66]. Obesity is a high-risk factor for precocious puberty [67]. According to a regional study in China, the prevalence of precocious puberty was 11.47% in girls and 3.26% in boys and was considerably higher in overweight (27.9%) and obese (48.0%) girls than in normal-weight girls (8.7%) [68]. A recent retrospective study confirmed a strong association of obesity with precocious puberty, with more pronounced effects in girls [10], which was consistent with other reports [66, 68, 69]. A theory of bidirectional effects between obesity and precocious puberty has prevailed [67]. Chinese data supported that early puberty in girls increased the risk of obesity [70]. Therefore, the authors propose that precocious puberty is an emerging obesity-related metabolic disease due to its metabolic outcomes in addition to consequent short final adult height and psychological disorders for children [71]. Notably, the current growth charts for evaluating growth and related disorders do not consider the potential impacts of puberty on the growth and development of Chinese children and adolescents. Thus, Pu et al. published growth curves for boys and girls in different Tanner phases after a large-scale multicenter investigation in China, which are critical to accurately assess the age-specific height and weight at different Tanner stages [72].

**Fig. 1:**
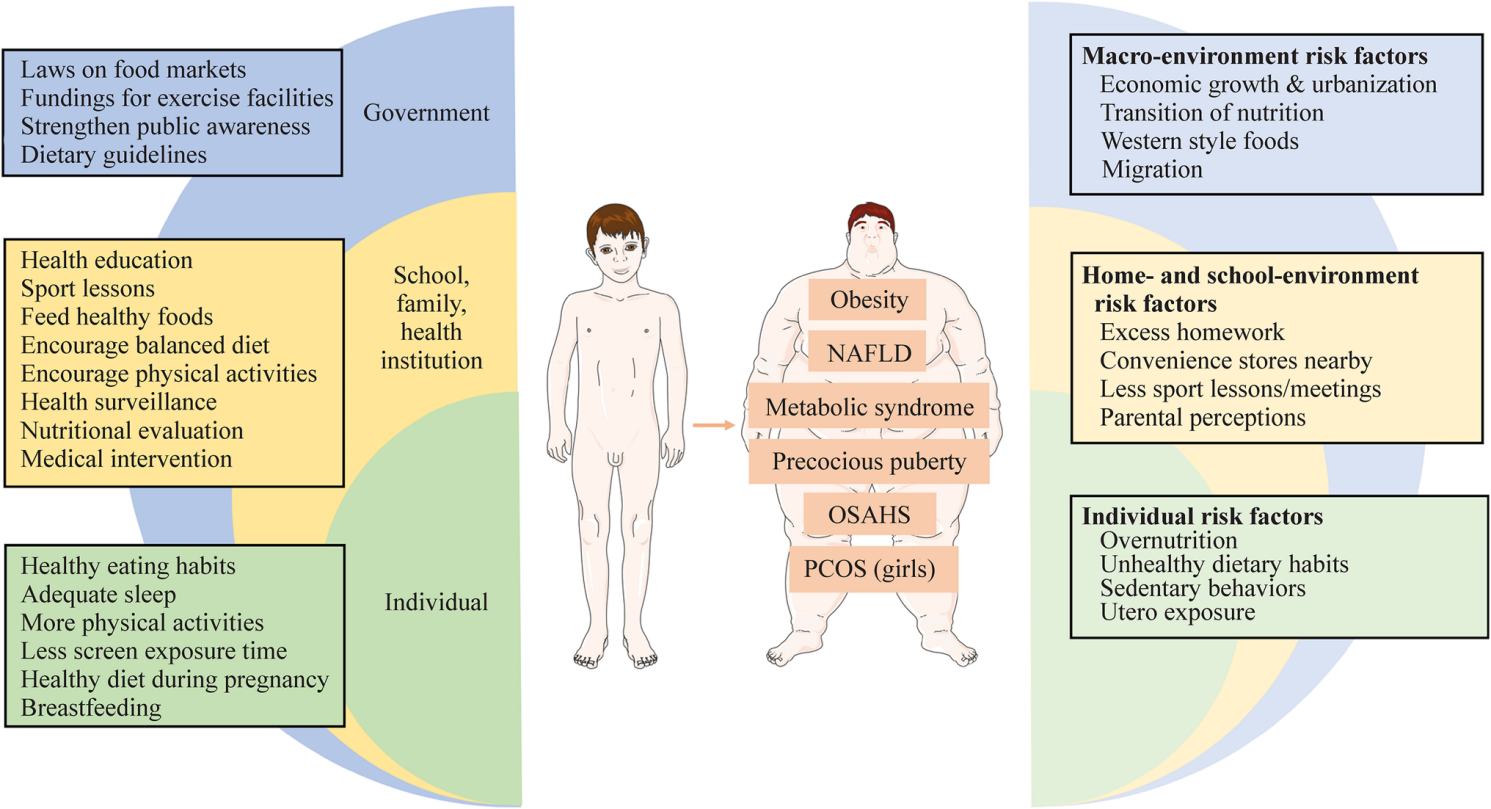
High-risk factors for childhood obesity and obesity-related disorders and measures for disease control and prevention accordingly. The right part of the figure presents high-risk factors for obesity. The left part of the figure specifies measures taken by governments, schools, families and health institutions. Individual actions are also highlighted. The middle part summarizes obesity-related metabolic diseases. *NAFLD* nonalcoholic fatty liver disease, *OSAHS* obstructive sleep apnea/hypopnea syndrome, *PCOS* polycystic ovary syndrome

Obesity is also a high-risk factor for other disorders, including obstructive sleep apnea/hypopnea syndrome and polycystic ovary syndrome [73–76], which profoundly threaten the health of children and adolescents (Fig. 1). Great attention should be given to obesity-related metabolic disorders, particularly emerging precocious puberty.

## Discussion

After having an updated picture of the prevalence of obesity and overweight among Chinese children and adolescents, it is necessary to explore possible reasons to provide appropriate policies accordingly. A bio-socio-ecological framework covering national policy, social environment, individual lifestyle, and genetic factors has been proposed to illustrate the changing prevalence [9], and a similar network applies for the Chinese population [77].

### Environmental risk factors

Multiple factors contribute directly or indirectly to the high prevalence of overweight and obesity (Fig. 1). The globalization and liberalization of trade promote the availability of a wide variety and large quantities of foods [78]. According to the CNSSCH survey, China’s developing economy, accompanied by improved nutritional status, is positively correlated with children’s obesity [4]. Western-style and fast foods have flourished in China [79, 80], providing easy access to overnutrition and unhealthy food. Governmental policies can shape people’s health. The one-child policy was effective during 1979–2015, and there is evidence that single children are more likely to develop overweight or obesity than peers with siblings [81]. Additionally, national guidelines for the prevention of childhood obesity were not proposed until 2007, when the prevalence of obesity surged [82].

Migration is an emerging factor in the increasing prevalence of overweight and obesity [83, 84]. Based on the seventh population census in China, developed eastern coastal areas maintained a higher inflow migration rate in the twenty-first century [85]. Over 19 million people born in other provinces (mainly northeast and midwest areas) migrated to Zhejiang Province from 2010 to 2020, accounting for 13.54% of all migrants throughout the country [85]. Importantly, family migration has prevailed recently, and thus, more children migrate with their parents into inflow places. The number of children in primary schools in eastern areas increased dramatically from 10% in 2010 to 40% in 2019 [86]. It is presumed that these young migrants are risk factors leading to the rapidly increasing prevalence of overweight and obesity in eastern areas of China, as migrants have been reported to have a higher ratio of overweight and obesity than the local population [84, 87]. Nevertheless, large-scale investigations on the influence of migrant children should be considered considering great disparities in culture and lifestyle between eastern coastal areas and outflow areas.

Home- and school-related environmental factors are involved in the increasing prevalence of obesity and overweight among children, particularly those in primary schools. High academic burden from excess homework [88] and the sale of carbonated drinks in schools and nearby convenience stores are highly associated with increased obesity [89, 90]. School activities, such as sport meetings, are also associated with BMI [90]. Mothers who eat out more frequently were reported to give more pocket money to their children, and increased pocket money facilitates excess energy intake by children [91, 92]. From a view of Chinese cultural norms, excess body fat is thought to be a sign of healthy growth [25], particularly in younger children and boys [93], and the misperception of children’s body size by their caregivers, including grandparents and parents, contributes to children’s overweight and obesity [94, 95].

### Individual risk factors

With the transition from the traditional Chinese to a Western diet [38, 96–98], personal eating habits have become a direct contributor to increased obesity and overweight (Fig. 1). Processed foods are directly associated with increased BMI [99]. In a cross-sectional study, more than half of the young participants consumed sugar-sweetened beverages, and those consuming higher amounts were more likely to have abdominal obesity [100]. Children’s eating-out behaviors are also associated with obesity [92]. Inadequate sleep, physical inactivity and screen viewing time outside schools, which are closely linked to energy balancing, are strong contributors to child obesity [88, 101, 102]. In China, pregnant women have diets enriched in various nutrients and remain sedentary, which are considered beneficial for gestation [103]. However, maternal overnutrition and gestational weight gain are likely to be responsible for macrosomia [104, 105].

In this review, we highlight a recent stabilized trend of overweight and obesity among Chinese children and adolescents, which is consistent with studies from other countries [106, 107]. A flattened mean BMI is reported despite the increase in the global age-standardized prevalence of obesity from 0.7% and 0.9% in 1975 to 5.6% and 7.8% in 2016 for girls and boys, respectively [108]. A series of studies relying on national surveys showed that the prevalence of obesity in American children at younger ages (< 11 years) stabilized or even declined, whereas it continued to increase among older children and adolescents aged 12–19 years [109–111]. However, there is contradictory evidence reporting no decline in the prevalence of obesity in children aged 2–19 years and a significant increase in children aged 2–5 years [112]. A more recent cohort study reported a worse obesity incidence than those in the past 12 years [113], highlighting the importance of national longitudinal design in monitoring the prevalence of obesity and overweight in children and adolescents. The slowing of the increase in BMI and obesity prevalence was also reported in Chinese adults according to the CCDRFS program [114]. More surveillance is needed to monitor and consolidate new trends in the prevalence of overweight and obesity in Chinese youths and adults in the future.

It would be fascinating to investigate the causes of the recent slight reversal of the long-term increases in overweight and obesity in Chinese children and adolescents. Government policies are the major tools in managing the burden caused by overweight and obesity in China. Since 2000, many official policies have been implemented, which highlight the importance of exercise, obesity surveillance, and healthy food intake, as reviewed elsewhere [77]. The protective effects of breastfeeding against overweight and obesity in younger children have been reported [115–117], and both breastfeeding rate and duration have increased rapidly in the past decade [118, 119]. A possible reason for the declining trend of obesity or overweight in girls is the self-evaluation of their body image. A number of girls perceive themselves as overweight, which leads to deliberate actions to lose weight, although many of them actually have normal weight [120, 121].

The problem remains challenging owing to a large percentage of obesity and overweight in the Chinese population, which can easily worsen in the future. According to Wang et al., 15.6% of preschool children and 31.8% of school-age children are projected to be overweight or obese in 2030 [77]. In addition, obesity-related metabolic disorders among Chinese children and adolescents are expected to increase the disease burden on the healthcare system. The intensification of the obesity epidemic by COVID-19 must not be ignored. A review article evaluated the lifestyle of the younger generation worldwide and concluded that COVID-19 restrictions led to unhealthy food choices, increased food intake, and reduced outdoor physical activities [122]. Consistently, the COVID-19 lockdown increased the prevalence of obesity and overweight in Chinese children at preschool and school age by changing their lifestyle, such as reducing physical activities and increasing screen-viewing time [31–34]. Undoubtedly, since the beginning of 2020, the COVID-19 outbreak has induced a sharp increase in the prevalence of obesity and overweight [37]. Thus, urgent precautions are imperative for evaluating the long-term effects of COVID-19 on the prevalence and management of obesity among Chinese children and adolescents.

### Conclusions and strategies for the prevention and management of childhood obesity

The prevalence of overweight and obesity among Chinese children and adolescents has been increasing, as reported by CNSSCH-, CHNS-, and CNNS-based studies (Table 1). Children in primary schools need lifestyle management, as they have a higher prevalence than older children and adolescents [5, 13 15]. The prevalence in boys gradually slows down, and that in girls appears to decrease [13]. Although a persistently higher prevalence of obesity exists in northern areas, the trend is not significant in recent years; meanwhile, the prevalence in eastern areas is surging [13]. The increase in obesity-related complications makes it challenging to address the burden of obesity [6, 10, 44, 45, 66, 68, 123]. Additionally, COVID-19 restrictions have worsened the epidemic of obesity in Chinese children and adolescents [31–34]. These could be attributed to the factors at national and social levels (e.g., transition of nutrition) [3, 4] and migration [85, 86]. Less healthy home and school environments, as well as personal behaviors, are more direct contributors, particularly to children in primary schools. Thus, dynamic surveillance needs to continue, and appropriate measures for disease prevention and management are imperative.

Several Chinese national policies and guidelines have been implemented since 2000, aiming to improve children’s health [77]. In October 2016, the State Council published the Health China 2030 Plan, aiming to establish a comprehensive system of health promotion and services [124]. In July 2019, a national health promotion committee under the State Council was organized and issued the Healthy China Program (2019–2030) [125]. This program proposed a variety of initiatives for the advancement of Chinese health status and disease control and prevention. In this program, children in primary and middle schools are required to have physical activities inside schools for > 1 hour per day. Additionally, health education curricula are to be introduced in all schools, and child health care experts are employed in over 70% of the schools. Activities outside schools, including sleeping and screen viewing time, are discussed for school students. In October 2020, six Chinese government agencies published “The Plan of Obesity Control and Prevention in Children and Adolescents” [126]. This plan aims to reduce the prevalence of obesity according to its different epidemic levels. The respective annual increases in regions with a high, intermediate, and low prevalence are 20%, 30%, and 40% of the baseline from 2020 to 2030. Zhejiang Province, with an intermediate prevalence, merits more attention considering the rapid increase in recent years. Notably, cooperation relying on internet platforms among the government, families, schools and health institutions was encouraged for better management of body weight and promotion of healthy growth and development in children and adolescents (Fig. 1).

Reducing energy intake and increasing energy expenditure without adverse influence on children’s growth and development are effective therapies for obesity [127]. The amount of energy intake for children with normal, overweight, and obese BMI at different ages has been tailored in the Nutritional Guidelines for Weight Management of school-aged children, released by the Chinese Nutrition Society in June 2021 [128]. Children are recommended to eat a balanced diet according to their age, sex, and level of physical activity. Nutritionists can help decide energy intake for obese and overweight children aged 6–12 years, while medical doctors can evaluate the balance between weight loss and normal development for obese or overweight children aged 13–17 years, particularly those who have early puberty onset. Two national policies published in the same year specified the work of different aspects and emphasized early diagnosis and intervention [129, 130]. The effectiveness of these policies and guidelines in the control and prevention of obesity among Chinese children and adolescents needs to be assessed by scientific evidence in the future.

## Data Availability

All data collected or interpreted during this study are included in this manuscript.
